# Nurse retention in Europe: why economic evidence matters

**DOI:** 10.1016/j.lanepe.2026.101822

**Published:** 2026-08-25

**Authors:** Claudia B. Maier, Marjorie Opuni, Julia Köppen, Carolin Gurisch, Matthias Wismar, Howard Catton, Margrieta Langins

**Affiliations:** a Department of Health Services Research and Nursing Science, School of Public Health, Bielefeld University, Bielefeld, Germany; b Independent Health Economist, Lausanne, Switzerland; c Department of Health Care Management, Technical University of Berlin, Berlin, Germany; d European Observatory on Health Systems and Policies, Brussels, Belgium; e International Council of Nurses, Geneva, Switzerland; f WHO Regional Office for Europe, Copenhagen, Denmark

**Keywords:** Personnel retention, Personnel turnover, Nurses, Costs and cost-analysis, Europe

## Abstract

Europe faces a health workforce crisis with nurses comprising the largest occupational group affected. International organisations argue that nurse retention should be the priority response. However, Europe lacks economic evidence to inform evidence-based action on retention. A research gap exists with regard to European studies that quantify nurse turnover costs, provide comparative cost data across countries, or evaluate the cost-effectiveness of retention initiatives. Other high-income countries have measured turnover costs, demonstrating both the feasibility of such analysis and the financial magnitude of the problem. Yet these findings cannot be transferred to European health systems due to differences in clinical practice, payment and other system characteristics, nurse skill-mix, and scope-of-practice. This Viewpoint addresses why turnover cost estimates from other regions cannot be applied to European health systems and what European hospital leaders, workforce planners, and policymakers could do with economic evidence they do not currently have. It calls for a European research agenda that prioritises comparative economic evidence on nurse retention to build the investment cases that decision-makers need. Economic evidence alone will not secure retention investments, but it is difficult to make the case for them without it.

## Introduction

The World Health Organization (WHO) has called for action to address Europe’s health workforce crisis.^1^, ^2^, ^3^ The 2024 Belgian Presidency of the European Union Council placed the health workforce at the centre of its agenda.^4^ While there is limited research on its magnitude, a recent analysis projects the shortage to be approximately 950,000 healthcare workers across Europe in 2030,^5^ with nurses comprising the largest occupational group affected.^6^ Over one-quarter of European nurses are aged 55 or older and expected to retire within the decade, while declining interest among young professionals compounds recruitment challenges.^6^ Burnout is widespread, affecting an estimated 30% of nurses in Europe.^7^^,^^8^ Intent to leave is high, with between 12% and 64% of hospital nurses across Europe intending to leave their current positions.^7^^,^^9^ The consequences for patients are measurable, with recent European evidence linking nurse intention to leave and heavier workloads with increased inpatient hospital mortality.^10^

International organisations have identified retention as the priority response.^3^^,^^11^ It has been argued that retention should take precedence over recruitment, given the training timelines required to produce new nurses and the ethical concerns surrounding international recruitment from countries with more fragile health systems.^3^ Reactivation, which draws back health workers who have left for other sectors, taken career breaks, or whose qualifications are not recognised, is a further strategy that remains neglected in European countries.^4^ Germany estimates that hundreds of thousands of trained health workers are employed outside the health sector.^4^ Yet European countries are managing this workforce challenge amid a lack of rigorous data. A recent policy brief noted that current workforce data systems “provide little information relevant to retention” with no internationally comparable data on key indicators and reporting discrepancies that complicate even basic surveillance.^11^

Nursing retention across European healthcare systems is also being addressed without rigorous economic evidence. We conducted a comprehensive search with no language restrictions in Medline (via PubMed), CINAHL, and Google Scholar in December 2025 using controlled vocabulary and free-text terms for nurses, nursing, turnover, retention, cost, and economic evaluation. We looked for peer-reviewed articles that reported per-nurse turnover costs in high-income countries and then narrowed the search to European countries, including all European Union (EU) member states. The most recent nurse turnover costing study we found in Europe dates back to 1996 and was conducted in the United Kingdom.^12^

Without costing studies and economic analyses in Europe that translate changes in turnover into budgetary impacts, and without effectiveness studies that show which interventions reduce turnover and improve retention, nurse retention investments struggle to secure funding amid constrained budgets.^13^^,^^14^ Proposals to improve nurses’ working conditions remain generic assertions that retention is cheaper than recruitment rather than quantified options. Without cost data, it is hard to defend nurse-centred reforms in budget negotiations and impossible to track whether promised savings from reduced turnover materialise. The case for the importance of economic evidence in nursing managerial and policy decisions is not new.^13^^,^^15^ The continuing absence of such data in Europe suggests funding constraints, but may also stem from broader structural and political barriers. This Viewpoint explains why cost estimates from other regions are not transferable to European health systems. It lays out what European hospital leaders, workforce planners, and policymakers could do with economic evidence they currently lack. It calls for a European research agenda of rigorous studies that would generate this evidence.

## Learning from other regions

While there is limited research on the cost-effectiveness of retention interventions globally, other high-income regions have measured turnover costs, demonstrating both the feasibility of such analysis and the financial magnitude of the problem.^13^^,^^16^ Reviews of international evidence outside Europe document substantial economic impacts of nurse turnover, though the evidence base is heavily concentrated in North America and Australasia.^16^, ^17^, ^18^ The United States (US) leads in this area, with NSI Nursing Solutions producing annual reports documenting that the average cost of turnover for a single bedside registered nurse has reached approximately $60,000, with individual hospitals facing losses in the millions of dollars annually.^19^ These reports quantify how changes in turnover rates translate into specific budgetary impacts, giving hospital executives concrete data for workforce planning and investment decisions.

One widely used approach for measuring these costs is the Nursing Turnover Cost Calculation Methodology.^20^^,^^21^ The methodology recognises that costs begin before a nurse formally resigns or retires (pre-notice disengagement affects productivity), continue through separation and the vacancy period, extend through recruitment and onboarding, and persist through orientation until the new hire reaches the productivity level of the departed nurse. It decomposes total costs into direct components (advertising, recruitment, onboarding, orientation and training, temporary cover) and indirect components (productivity losses, loss of organisational knowledge). This and other methodologies underpinning costing studies conducted in North America and Australasia provide European countries with useful templates.^14^^,^^16^^,^^18^, ^19^, ^20^, ^21^, ^22^

## Why cost estimates do not transfer

Cost estimates generated in different country contexts are not readily transferable. When researchers applied standardised methodology across four countries with relatively similar income levels, per-nurse turnover costs varied considerably.^23^ Variation despite methodological consistency suggests that turnover cost estimates reflect context-specific factors.

Health economists generally recommend using local cost data, arguing that estimates from elsewhere have limited transferability.^24^ Because clinical practices, payment systems, incentives, and the scope to reallocate resources differ across settings, cost estimates do not transfer well across locations and countries. An overview of cost transferability within the EU identified five sources of cost variation between countries: definition of the unit of activity, quantity and quality of resource inputs, average price levels, currency exchange rates, and cost-accounting methodology.^25^

Cost transferability challenges begin with basic definitions. What constitutes turnover varies across settings. Some systems count only voluntary resignations while others include retirements, dismissals, and internal transfers.^23^^,^^26^ Vacancy periods are defined differently based on when positions are officially posted, when offers are made, and when new hires are considered fully operational.^23^ These differences in definitions complicate comparisons of turnover rates across countries.

European researchers do not need to develop costing frameworks from scratch, since existing nurse turnover methods can be adapted to different contexts. What is needed are nurse turnover cost estimates generated across several European health systems. Even with standardised methodologies such as the Nursing Turnover Cost Calculation Methodology, researchers still have to make context-specific decisions about which cost components to include, how to measure productivity losses, and how to allocate overhead expenses (e.g., administration, management, facility maintenance).^15^^,^^16^ Accounting treatment of orientation costs, productivity losses, and overhead allocations varies across institutional settings.^17^^,^^23^ What appears as a recruitment cost in one setting may be absorbed as general administrative overhead in another. Some studies on nurse turnover costs have used other methods entirely.^16^, ^17^, ^18^

Clinical practices and workforce composition differ across countries. How care is organised, who performs which tasks, and what skill mix is deployed all affect turnover costs. Moreover, interprofessional workforce dynamics, such as team composition and teamwork configurations, add further analytical complexity.^27^ Different professional mobility patterns, education and training requirements, and scope of practice regulations mean that replacing a departing nurse involves different processes and resources across systems. A further source of variation is the baseline itself, including performance, productivity, workflows and efficiency as well as quality and person-centred care. Task-shifting and evolving models of care mean productivity varies across countries and changes within the same system over time.

Labour market structures add another source of variation. The availability, cost, and regulatory frameworks for temporary replacement staff differ across countries. Many European countries have strong collective bargaining frameworks with mandated notice periods, dismissal procedures, and consultation requirements that affect both termination costs and typical vacancy durations.^28^ What constitutes a feasible response to a vacancy in one setting may not be available or legal in another.

Price differences are perhaps the clearest reason costs do not transfer. US health prices are well above the OECD average, and these price differentials have remained stable despite major health system reforms.^29^ The US spent approximately twice as much on healthcare despite similar utilisation patterns, with higher prices for labour, goods, pharmaceuticals, and administrative costs driving the difference.^30^ US nurse and other professional salaries and administrative costs are substantially higher than in comparison countries.^30^ Nurse turnover cost calculations directly incorporate salary levels, administrative system costs, and replacement expenses.

## Building investment cases

Decision-making tools that demonstrate whether proposed investments are worth making, commonly termed “business cases” in healthcare management, allow decision-makers to evaluate workforce investments against competing priorities using common financial metrics.^13^ These investment cases answer the questions that hospital leadership teams and governing bodies ask: what it will cost to implement, what financial returns it will generate, over what timeframe, and what evidence supports these projections. Although economic evidence is of relevance to hospitals, particularly those that rely on business case logic, its role in decision-making likely differs across European health system contexts. A study across 10 European countries, including tax-funded and social-health insurance systems, found that hospitals vary considerably in their autonomy and decision-making structures, shaped by hospital governance (centralised vs. decentralised), bargaining arrangements (individual vs. collective), and institutional as well as political priorities.^31^

For nurse retention in hospitals and other healthcare settings, building these value propositions requires quantifying both costs and possible savings.^13^ This Viewpoint focuses primarily on hospital nursing, where research on turnover costing is currently most developed internationally, although retention challenges also exist in primary care, community settings, and long-term care. Hospitals first need to establish what nurse turnover currently costs their organisation (the losses from continuously replacing departing staff), then quantify what specific retention interventions would cost to implement, what reductions in turnover (and other key workforce- and patient-related outcome data) those interventions would achieve, and what financial savings, if any, would result.

Evidence from hospitals that have built investment cases shows why turnover costs are central to these justifications. One analysis of US hospitals pursuing quality designations showed that nurse vacancy reduction accounted for the majority of quantifiable savings, with nurse-sensitive patient safety outcomes (e.g., pressure ulcers, falls) contributing the remainder.^32^ Even after accounting for programme preparation costs, large net positive returns emerged in the first year.^32^

The absence of economic evidence may affect workforce planning for individual facilities as well as country health systems. European hospital leaders, country workforce planners, and policymakers lack evidence to compare different workforce strategies. They cannot determine whether investing in retention programmes, improving working conditions, scaling up education, or expanding international recruitment represent cost-effective strategies. The International Council of Nurses’ 2022 call for “building the business case” for retention^15^ cannot be answered with European data because the cost evidence to build such cases does not exist.

In 2026, Europe still lacks recent, comparable turnover cost estimates and a sustainable programme to generate and update such evidence. European hospitals face a workforce crisis documented in the same policy language of urgency,^2^^,^^3^^,^^7^^,^^11^ yet lack the economic evidence needed to justify solutions.

## Call for a European research agenda

Europe needs systematic nurse turnover cost research adapted to its diverse health system contexts. A methodology exists that researchers could build on to reflect different salary structures, administrative systems, training models, and replacement strategies.^13^^,^^20^^,^^21^ Yet, adaptation is not a matter of adjusting figures from other contexts. It requires primary data on how European healthcare system characteristics (payment models, workforce compositions, organisational structures, labour market dynamics) shape different cost profiles.^25^ Multi-country comparative studies within Europe would document cost variation across European health systems and identify where costs can and cannot be transferred. These studies could group countries into clusters according to health systems financing (Beveridge, Bismarck), hospital governance mechanisms (centralised vs. decentralised), and workforce planning (centralised vs. decentralised), for example.^31^

Turnover cost research should be paired with economic evaluations of retention interventions. Hospitals need to know not only what turnover costs currently, but what specific retention programmes cost to implement, what savings they generate, and what return on investment they deliver.

A European nurse turnover cost research infrastructure would allow collaborations to develop standardised turnover definitions, common costing approaches across different contexts, routine workforce data collection and link it with workforce- and patient-related outcome data. A first step could be a cross-country feasibility study across selected countries and hospital systems to identify turnover cost estimates within European contexts. Establishing an interdisciplinary network of health economics, nursing, workforce, and health systems researchers, including the European Observatory on Health Systems and Policies and the WHO Regional Office for Europe, would provide the foundation for costing estimates, comparative analyses, and economic evaluation of retention interventions. Cost-effectiveness analyses of retention interventions could help identify which approaches work best for different hospital types, workforce compositions, and regional labour markets.

European hospital leaders could take retention investment proposals to financing bodies with detailed supporting analyses. Financing bodies could evaluate retention programmes against competing priorities using comparable financial metrics. They could assess returns on retention investment using the same frameworks applied to other health system investments. This shifts nurse retention from moral imperative to justified investment.

At the same time, generating economic evidence does not mean it automatically or easily translates into management or policy decisions. There is substantial evidence on the evidence-to-practice and evidence-to-policy gaps.^33^, ^34^, ^35^ Multiple context-specific factors influence decision-making.^33^, ^34^, ^35^ In particular, the role of politics and stakeholders is key, including interest group politics, bureaucratic politics, budget politics, leadership politics, beneficiary politics, and external actor politics.^33^ Important factors include dissemination formats and channels tailored to the needs of decision-makers, timing, stakeholder role and involvement, and the political economy (budget cycles, institutional inertia, etc.), among others.^35^ Policy advocacy and knowledge brokering are needed to institutionalise the use of evidence and mediate interest group politics.^33^^,^^35^

Advancing this agenda requires commitment from funders, ministries, and hospital systems. European research funding bodies should treat turnover costing studies as a priority for sustained investment. Programmes like Horizon Europe could prioritise generating policy-relevant economic evidence for retention investment decisions, with part of the funding directed at research on dissemination and knowledge brokering strategies. National health ministries could proactively require economic evaluation alongside implementation of major retention initiatives. This would create both demand for and supply of rigorous cost data. Hospitals and other healthcare facilities will need to invest in the costing infrastructure (data systems, analytic capacity, finance-workforce collaboration) required to generate and apply turnover cost evidence internally.

Government Chief Nursing Officers, professional nursing organisations, other stakeholders, and researchers have a role in championing this agenda. European nursing bodies can insist investment cases rest on European evidence rather than imported estimates. Health economics research networks could treat nurse turnover costing as a priority area requiring methodological development and multi-country coordination.

To conclude, Europe has the research capacity to measure nurse turnover costs, and an urgent need to retain its nursing and health workforce in clinical care. What it does not yet have is strong economic evidence on which to base retention decisions. Generating this evidence will not guarantee retention investments, as workforce policy decisions are also shaped by political, institutional, and fiscal constraints. However, the limited availability of economic evidence on turnover may represent an additional barrier to policy uptake in resource-constrained and politically competitive environments. Producing rigorous, cross-country research to inform investment cases and policy-making is critical to making retention investments justifiable.

## Contributors

CBM and MO contributed equally to the Viewpoint and share first authorship. CBM and MO conceptualised the content and structure of this Viewpoint. CBM and MO were responsible for the drafting and subsequent revisions of the manuscript. JK, CG, MW, HC and ML provided important input to the concept of this Viewpoint, reviewed and revised the manuscript. All authors agreed on the final version to be published and to be accountable for all aspects of the work. CBM and MO made the final decision to submit the Viewpoint for publication after agreement was reached with all co-authors.

## Declaration of interests

CBM, JK, CG, MW, HC, ML declare coverage of travel costs through EU-funded projects or other non-profit organisations to attend conferences and speak at meetings (no personal fees). CBM and JK declare financial support from the German Ministry of Health and Ministry of Education and Research as well as Bosch Health Campus to Bielefeld University, where they are employed. CG declares financial support from Bosch Health Campus as part of her doctoral studies at Technische Universität Berlin. CBM and JK have received financial support paid to Technische Universität Berlin, where CBM was previously employed and JK is currently employed. MW declares financial support from the European Commission (BeWell, HEROES Joint Action) to his employer. MW and ML are employed by WHO. CBM has had technical consultancy contracts with WHO and ICN. The authors affiliated with WHO are solely responsible for the views expressed in this Viewpoint; they do not necessarily represent the decisions or policies of WHO.
